# Complete genome sequencing of *Agrobacterium leguminum* strain EL101 isolated from *Khaya senegalensis* tree bark using nanopore technology

**DOI:** 10.1128/mra.00298-24

**Published:** 2024-06-27

**Authors:** Wai Keat Toh, Han Ming Gan, Hann Ling Wong

**Affiliations:** 1 Department of Biological Science, Faculty of Science, Universiti Tunku Abdul Rahman, Kampar, Perak, Malaysia; 2 Patriot Biotech Sdn. Bhd, Bandar Sunway, Selangor, Malaysia; University of Strathclyde, Glasgow, United Kingdom

**Keywords:** *Agrobacterium leguminum*, complete genome sequence

## Abstract

We report the complete genome sequence of *Agrobacterium leguminum* strain EL101, isolated from the tree bark of *Khaya senegalensis* in Kampar, Perak, Malaysia, obtained using Q20+ Nanopore Sequencing chemistry. The assembled genome has a total length of 5,324,685 bp, comprising a circular chromosome, a linear chromid, and two non-Ti circular plasmids.

## ANNOUNCEMENT

Agrobacteria are commonly found in soil microflora, with the majority being non-phytopathogenic (1). Recently, a novel species, *Agrobacterium leguminum*, was first isolated from the nodules of *Phaseolus vulgaris* grown in southeastern Spain (2).

To isolate strain EL101, 30 g of tree bark samples exhibiting signs of infection was collected from three individual *Khaya senegalensis* located in the Kampar area (4°20′11.7″N 101°08′47.3″E). The samples were initially surface-sterilized with 10% commercial bleach, washed thrice with sterile distilled water, ground with a pestle, and then immersed in a 0.85% saline solution followed by vortexing for 5 mins. The suspension was briefly centrifugated, and the supernatant was plated on *Agrobacterium* selective 1A medium supplemented with 80 µg/mL of K_2_TeO_3_ (3). Following incubation, typical domed, glistening morphologies of agrobacteria were observed. A pure culture was obtained by subjecting a single colony to three consecutive subcultures by plate streaking.

Genomic bacterial DNA was extracted from the culture grown in 5 mL of nutrient broth at 28°C overnight with 220 rpm agitation using the NucleoSpin Microbial DNA Mini Kit for DNA (Machery Nagel, Düren, Germany) following the manufacturer’s instructions. For Oxford Nanopore Technologies (ONT) sequencing, 500 ng of the unsheared DNA without size selection was directly used as the template for the ONT Ligation Sequencing Kit (SQK-LSK114). Sequencing was performed on the ONT MK1b device using Flongle R10.4.1 flow cells for 24 hrs. Basecalling and adapter-trimming were performed using Dorado v0.5.1 (https://github.com/nanoporetech/dorado), trained on the dna_r10.4.1_e8.2_400bps_sup@v4.3.0 basecalling model, generating 177,430 reads with a N50 value of 11,235 bp. The raw reads (no error correction performed) were subsequently assembled *de novo* using Flye 2.9 (4). Circular genomes were identified during Flye assembly based on the presence of overlapping ends, trimmed to remove overlap, and reported as it is without additional rotation. Validation of the completeness of an assembled linear contig as a linear chromosome (chromid) was based on a BLASTN search against known linear chromids of *Agrobacterium* spp. Default parameters were used for all software unless otherwise specified.

The complete genome consists of 5.32 Mb (59.78% GC; N50 of 2,970,114 bp) and was assembled into four contigs: a circular chromosome (chromosome I; 1,979,384 bp; 60.0% GC), a linear chromid (chromosome II; 2,970,114 bp; 59.8% GC), and two circular non-Ti plasmids (pEL101-131; 131,537 bp; 58.7% GC, and pEL101-243; 243,650 bp; 58.8% GC). Analysis using FastNI v1.34 (5) showed that strain EL101 exhibits the highest average nucleotide identity (ANI) of 97.48% and genome coverage of 78.2% to *A. leguminum* (previously *Agrobacterium deltaense*) YIC4121 (GCF_002008205.1) (6). Additionally, the ANI relative to the type strain *A. leguminum* MOPV5^T^ (GCA_015704895.1) was 95.94% with a genome coverage of 83.78% (2). Annotation by NCBI Prokaryotic Genome Annotation Pipeline v6.6 (7) identified 12 rRNA genes, 53 tRNA genes, 4,874 protein-coding genes, and 106 pseudogenes. It is noteworthy that only the *vir*B operon was detected, suggesting that strain EL101 does not possess the genomic potential to induce tumors in plants due to the absence of other essential virulence genes required for a functional Type IV secretion system.

## Data Availability

Genome sequences of EL101 were deposited in GenBank under accession numbers CP143885 (chromosome I), CP143886 (chromosome II), CP143887 (plasmid pEL101-131), and CP143888 (plasmid pEL101-243). Nanopore reads were deposited in the Sequence Read Archive under accession number SRR27795679.
